# Association of Race/Ethnicity and Other Demographic Characteristics With Use of Physical Restraints in the Emergency Department

**DOI:** 10.1001/jamanetworkopen.2020.35241

**Published:** 2021-01-25

**Authors:** Ambrose H. Wong, Travis Whitfill, Emmanuel C. Ohuabunwa, Jessica M. Ray, James D. Dziura, Steven L. Bernstein, Richard Andrew Taylor

**Affiliations:** 1 Department of Emergency Medicine, Yale School of Medicine, New Haven, Connecticut; 2 Department of Pediatrics, Yale School of Medicine, New Haven, Connecticut

## Abstract

This cross-sectional study assesses the association of race/ethnicity and other demographic factors with risk of receiving physical restraint during an emergency department (ED) visit.

## Introduction

Patient encounters in the emergency department (ED) commonly include symptoms of agitation, defined as excessive psychomotor activity leading to aggressive and violent behavior, that can cause serious injuries to staff and patients.^1^ A recent study^2^ at an urban level 1 trauma center found that 2.6% of all ED visits involved agitation. Physical restraints are routinely used and indicated during management of agitation in situations in which danger is imminent or when de-escalation measures have failed. However, physical restraints are associated with minor injuries to more serious complications, including apnea and cardiac arrest.^3,4^ This association is especially important given that ED patients with behavioral disturbances often represent socioeconomically distressed populations,^5,6^ thus placing them at risk for differential treatment. This study aimed to assess factors that may be associated with a higher risk of receiving physical restraint during an ED visit. We hypothesized that socioeconomic and demographic factors would have significant associations with the odds of restraint use.

## Methods

We conducted a cross-sectional study of all adult (age, ≥18 years) patient visits to the ED at 3 hospitals within the Yale-New Haven Health System in Connecticut from January 2013 to August 2018. Our primary outcome was the presence of a physical restraint order in the electronic health record during an ED visit. The study was approved by the Yale University human investigation committee. Informed consent was waived because the study posed minimal risk to individuals. This study followed the Strengthening the Reporting of Observational Studies in Epidemiology (STROBE) reporting guideline.

We conducted a descriptive analysis of the data and used a generalized linear mixed model with a binary logistic link for the presence of a restraint order. Variables in the model included demographic characteristics (collected during intake questionnaire), which consisted of sex, race/ethnicity, age, insurance status, alcohol use, illicit drug use, and homelessness (Table 1). Visit characteristics consisted of discharge diagnosis, chief concern, use of the Emergency Severity Index level at triage, arrival time of the day, number of prior ED visits, and number of prior admissions to an inpatient unit. Our model incorporated nesting by site and patient. All tests were 2-tailed, and *P* < .05 was considered statistically significant. Analyses were conducted using SPSS statistical software, version 22.0 (IBM Corp).

**Table 1.** Demographic and Visit Characteristics of Patients Visiting the ED by Presence of a Physical Restraint Order in the Electronic Medical Record, January 2013 to August 2018

Characteristic	Restraint use^a^	
	No (n = 719 327)	Yes (n = 7090)
ED type		
Suburban	93 219 (13.0)	31 (0.4)
Community	207 119 (28.8)	917 (12.9)
Urban	418 989 (58.2)	6142 (86.6)
Sex		
Male	321 706 (44.7)	4597 (64.8)
Female	397 620 (55.3)	2494 (35.2)
Age, mean (SEM)	49.61 (0.02)	45.63 (0.22)
Race		
Asian	7106 (1.0)	36 (0.5)
Black or African American	202 943 (28.2)	2041 (28.8)
White	383 979 (53.4)	2852 (54.3)
Other^b^	125 299 (17.4)	1161 (16.4)
Ethnicity		
Hispanic or Latino	120 325 (16.7)	1042 (14.7)
Non-Hispanic Black or White	593 822 (82.6)	5981 (84.4)
Unknown	5180 (0.7)	67 (0.9)
Insurance status		
Private	238 742 (33.2)	1251 (17.6)
Medicaid	248 958 (34.6)	3486 (49.2)
Medicare	156 041 (21.7)	1548 (21.8)
Self-pay	3661 (0.5)	80 (1.1)
Other	71 925 (10.0)	725 (10.2)
Illicit substance use		
No	526 439 (73.2)	3905 (55.1)
Yes	106 809 (14.8)	2604 (36.7)
Not asked	86 089 (12.0)	581 (8.2)
Alcohol use		
No	391 708 (54.5)	3067 (43.3)
Yes	268 727 (37.4)	3641 (51.4)
Not asked	58 892 (8.2)	382 (5.4)
Homeless		
No	716 521 (99.6)	6926 (97.7)
Yes	2806 (0.4)	164 (2.3)
Discharge diagnosis^c^		
Medical	566 460 (78.7)	4854 (68.5)
Psychiatric	228 625 (31.8)	4141 (58.4)
Alcohol or drugs	72 782 (10.1)	2103 (29.7)
Cognitive or neurologic	62 106 (8.6)	1201 (16.9)
Trauma	49 018 (6.8)	1045 (14.7)
Chief concern^c^		
Medical	326 633 (45.3)	1057 (14.9)
Psychiatric	20 543 (2.9)	1321 (18.6)
Alcohol or drugs	16 142 (2.2)	1430 (20.2)
Cognitive or neurologic	10 003 (1.4)	502 (7.1)
Trauma	52 864 (7.3)	409 (5.8)
Emergency Severity Index level^d^		
1	7045 (1.0)	570 (8.0)
2	202 233 (28.1)	5692 (80.3)
3	315 031 (43.8)	760 (10.7)
4	161 067 (22.4)	61 (0.9)
5	33 951 (4.7)	7 (0.1)
Arrival time		
3 am to 6 am	41 152 (5.7)	492 (6.9)
7 am to 10 am	131 979 (18.3)	668 (9.4)
11 am to 2 pm	185 870 (25.8)	1438 (20.3)
3 pm to 6 pm	167 525 (23.3)	1707 (24.1)
7 pm to 10 pm	128 417 (17.9)	1707 (24.1)
11 pm to 2 am	64 384 (9.0)	1078 (15.2)
ED visits, mean (SEM)	3.31 (0.01)	7.5 (0.27)
Hospital admissions, mean (SEM)	0.83 (0.003)	1.22 (0.03)

Abbreviations: ED, emergency department; SEM, standard error of the mean.

^a^ Data are presented as number (%) of patients unless otherwise indicated. Percentages may not total 100% due to rounding.

^b^ The “other” group included American Indian or Alaska Native, Native Hawaiian, other Pacific Islander, and unknown categories.

^c^ Chief concerns and diagnoses were grouped into 5 categories in accordance with prior work regarding use of restraints in the ED.^5^

^d^ The Emergency Severity Index is a 5-level ED triage algorithm that provides clinically relevant stratification of patients into 5 groups from 1 (most urgent) to 5 (least urgent) on the basis of acuity and resource needs.

## Results

A total of 726 417 total ED visits occurred during the study period, of which 7090 (1%) had associated physical restraint orders. Of individuals with restraint orders during their visit, 4597 (64.8%) were male, 2494 (35.2%) were female, 2041 (28.8%) were Black or African American, 1042 (14.7%) were Hispanic or Latino, 5034 (71%) had Medicaid or Medicare insurance, and 164 (2.3%) were homeless. In our model, Black or African American individuals were more likely to be restrained than were White individuals (adjusted odds ratio [AOR], 1.13; 95% CI, 1.08-1.21). Hispanic or Latino individuals (AOR, 0.78; 95% CI, 0.70-0.88) had lower odds of being restrained compared with non-Hispanic individuals (Table 2). Female individuals (AOR, 0.75; 95% CI, 0.71-0.79) had lower odds of being restrained than male individuals, and patients with Medicaid (AOR, 1.55; 95% CI, 1.45-1.67) or Medicare coverage (AOR, 1.67; 95% CI, 1.54-1.82) had increased odds compared with patients with private insurance. Patients who were homeless (AOR, 1.35; 95% CI, 1.14-1.16) also had increased odds of restraint use.

**Table 2.** Odds of Receiving a Physical Restraint Order by Variable in a Logistic Regression Model

Characteristic	Adjusted OR (95% CI)	*P* value
Sex		
Male	1 [Reference]	NA
Female	0.75 (0.71-0.79)	<.001
Age	0.99 (0.98-0.99)	<.001
Race		
Asian	0.78 (0.56-1.09)	.15
Black or African American	1.13 (1.07-1.21)	<.001
White	1 [Reference]	NA
Other	1.11 (0.99-1.24)	.07
Ethnicity		
Hispanic or Latino	0.78 (0.70-0.88)	<.001
Non-Hispanic Black or White	1 [Reference]	NA
Unknown	1.83 (1.42-2.37)	<.001
Insurance status		
Private	1 [Reference]	NA
Medicaid	1.55 (1.45-1.67)	<.001
Medicare	1.67 (1.54-1.82)	<.001
Self-pay	1.55 (1.22-1.97)	<.001
Other	1.45 (1.31-1.60)	<.001
Illicit substance use		
No	1 [Reference]	NA
Yes	1.55 (1.47-1.65)	<.001
Not asked	1.13 (0.99-1.28)	.05
Alcohol use		
No	1 [Reference]	NA
Yes	1.13 (1.07-1.20)	<.001
Not asked	0.89 (0.77-1.04)	.14
Homeless		
No	1 [Reference]	NA
Yes	1.35 (1.14-1.16)	<.001
Discharge diagnosis		
Medical	0.63 (0.58-0.65)	<.001
Psychiatric	1.74 (1.64-1.85)	<.001
Alcohol or drugs	1.14 (1.07-1.21)	<.001
Cognitive or neurologic	1.30 (1.21-1.39)	<.001
Trauma	1.11 (1.03-1.19)	.005
Chief concern		
Medical	0.43 (0.40-0.46)	<.001
Psychiatric	1.42 (1.32-1.52)	<.001
Alcohol or drug use	2.48 (2.30 2.67)	<.001
Cognitive to neurologic	3.14 (2.84-3.48)	<.001
Trauma	1.09 (0.98-1.21)	.12
Emergency Severity Index level		
1	1 [Reference]	NA
2	0.25 (0.22-0.27)	<.001
3	0.04 (0.04-0.05)	<.001
4	0.006 (0.004-0.007)	<.001
5	0.003 (0.001-0.006)	<.001
Arrival time		
3 am to 6 am	1.38 (1.22-1.56)	<.001
7 am to 10 am	1 [Reference]	NA
11 am to 2 pm	1.18 (1.07-1.29)	.001
3 pm to 6 pm	1.34 (1.23-1.47)	<.001
7 pm to 10 pm	1.44 (1.31-1.58)	<.001
11 pm to 2 am	1.47 (1.33-1.63)	<.001
No. of emergency department visits	1.00 (1.00-1.00)	.39
No. of hospital admissions	0.96 (0.94-0.97)	<.001

## Discussion

Our study found significant associations between Black or African American race, male sex, non-Hispanic ethnicity, lack of private insurance, and homelessness and increased risk of being physically restrained during an ED visit. In addition, visits involving behavioral chief concerns, higher acuity, and later time of day at presentation were associated with higher odds of use of restraints. This study has limitations. Our cross-sectional design limited our ability to make causal inferences from the study results. Our work describes restraint use overall and does not identify inappropriate restraint use, which may be more salient.

The increased odds of physical restraint associated with demographic variables, particularly race/ethnicity, may reflect potential implicit and systemic bias regarding decisions to physically restrain patients as well as upstream systemic biases and social determinants of health that may influence the likelihood of patients experiencing these situations. Further work is needed to identify structural factors contributing to potential disparities in treatment and interventions to avoid further marginalization of disadvantaged individuals.
